# Effect of pH on the Stability of Dairy Beverages Stabilized with Soluble Soybean Polysaccharides

**DOI:** 10.3390/foods14213632

**Published:** 2025-10-24

**Authors:** Hongyang Pan, Xiaofang Chu, Shiwen Li, Zhaojun Wang, Jie Chen

**Affiliations:** 1 State Key Laboratory of Food Science and Resources, Jiangnan University, Wuxi 214122, China; 2 Analysis and Testing Center, Jiangnan University, Wuxi 214122, China; 3 Institute of Botany, Jiangsu Province and Chinese Academy of Sciences, Nanjing 210014, China; cxf1002@163.com; 4 Zhejiang Gengshengtang Ecological Agriculture Co., Ltd., Huzhou 313300, China

**Keywords:** soluble soybean polysaccharides, dairy beverage, pH, milk protein, particle size, ζ-potential

## Abstract

This study aimed to elucidate the effect of pH on the stability of soluble soybean polysaccharide (SSPS)-stabilized dairy beverages. A single-factor experimental design was employed using model systems containing 1.0% (*w*/*v*) protein and 0.4% (*w*/*v*) SSPS, with pH values adjusted from 3 to 7. System stability was comprehensively evaluated through centrifugation sedimentation rate, particle size distribution, ζ-potential, viscosity, and LUMisizer analysis. As pH increased from 3 to 7, the stability first decreased and then increased, showing the lowest stability at pH 5 and the highest stability at pH 6–7. At pH 5, large SSPS–protein aggregates formed due to the proximity to the isoelectric point (pI) of milk proteins, resulting in increased viscosity (6.83 mPa·s) and reduced ζ-potential (−5.8 mV). Conversely, at pH 6–7, strong electrostatic repulsion and steric stabilization led to small, uniformly dispersed particles and minimal transmittance change (<5%) in LUMisizer analysis. These findings clarify the stabilizing mechanism of SSPS and provide practical guidance for pH regulation in the formulation of dairy beverages.

## 1. Introduction

In recent years, formulated dairy beverages have become one of the fastest-growing categories in the dairy industry, driven by their balanced nutritional profile (including high-quality milk proteins, vitamins, and minerals), refreshing taste, and convenient consumption [1,2]. According to the *2024 China Dairy Industry Report*, the annual consumption of formulated dairy beverages in China has exceeded 3 million tons, accounting for 12.3% of total dairy product consumption. However, product stability remains the key bottleneck hindering quality improvement and shelf-life extension. During production, transportation, and storage, issues such as protein aggregation, particle sedimentation, and phase separation frequently occur, leading to deterioration of sensory quality and reduced consumer acceptance. It is estimated that stability-related problems result in annual economic losses exceeding 1 billion yuan in the industry [1,3].

The stability of dairy beverage systems is influenced by multiple factors, including protein concentration, stabilizer type and dosage, pH, and processing conditions. Among these, pH is particularly critical, as it directly affects the conformation and charge properties of milk proteins [4]. Caseins, which account for approximately 80–82% of milk proteins, have an pI of around pH 4.6. Near this value, the net surface charge of casein molecules approaches zero, reducing electrostatic repulsion and promoting aggregation and sedimentation [1,5]. Whey proteins, which account for about 20% of total milk proteins, have pIs in the range of pH 5.1–5.3 and exhibit similar stability fluctuations, further increasing the risk of system instability. Consequently, pH regulation to avoid the pI effect of milk proteins has become a key strategy for improving the stability of formulated dairy beverages. Near the pIs of caseins (pH ~4.6) and whey proteins (pH 5.1–5.3), the net surface charge of milk proteins approaches zero, which weakens electrostatic repulsion, promotes protein aggregation and sedimentation, and reduces system stability. Therefore, controlling pH to remain away from these values is critical for maintaining a uniform and stable beverage system.

Soluble soybean polysaccharide (SSPS), a natural plant-derived polysaccharide, possesses excellent water solubility, emulsifying properties, and charge-regulating capacity, and has therefore been widely applied as a stabilizer in dairy beverages in recent years [6]. Previous studies have demonstrated that SSPS can adsorb onto the surface of milk protein particles, thereby forming steric barriers or modulating surface charges to inhibit protein aggregation [7,8]. However, most existing research has focused on the stabilizing effect of SSPS under a single pH condition, typically around neutral pH (pH 6–7). Few studies have systematically examined SSPS-stabilized dairy beverages across the full pH range of 3–7, which is essential for understanding stability under conditions relevant to most formulated products. This study systematically investigates the interactions between SSPS and milk proteins across different pH values, focusing on the coordinated variation of stability-related indicators such as particle size, ζ-potential, and viscosity. These findings provide practical guidance for pH regulation in the industrial production of SSPS-stabilized dairy beverages [9,10].

Based on this rationale, the present study established a formulated dairy beverage model containing 1.0% protein and 0.4% SSPS to systematically investigate the influence of pH (3–7) on system stability and the underlying mechanisms using multiple analytical techniques, including centrifugation sedimentation rate, particle size distribution, ζ-potential, viscosity, and LUMisizer analysis. The findings provide a scientific basis for enhancing product stability and offer practical guidance for optimizing process parameters in industrial production.

## 2. Materials and Methods

### 2.1. Materials and Reagents

Soluble soybean polysaccharide (SSPS, CA 700, purity ≥ 92%, molecular weight ≈ 2.5 × 10^5^ Da) was purchased from Fuji Oil Co., Ltd. (Osaka, Japan). Pectin was obtained from Danisco Co., Ltd. (Copenhagen, Denmark). Skim milk powder (protein content 34.5% on a dry basis, fat content ≤ 0.5%) was supplied by Fonterra Co-operative Group (Auckland, New Zealand). Bovine serum albumin (BSA) was purchased from Sigma-Aldrich Trading Co., Ltd. (Shanghai, China). Dextran molecular weight standards were obtained from Aladdin Reagent Co., Ltd. (Shanghai, China). Citric acid, sodium citrate, copper sulfate, potassium tartrate, sodium hydroxide, and sodium carbonate (analytical grade) were purchased from Sinopharm Chemical Reagent Co., Ltd. (Shanghai, China). Ultrapure water (conductivity ≤ 10 μS/cm) was prepared in the laboratory.

### 2.2. Instruments and Equipment

The main instruments used in this study included an AH-BASIC high-pressure homogenizer (Lerongen Technology Co., Ltd., Shanghai, China) for beverage preparation; a SIGMA 3K15 refrigerated centrifuge (SIGMA, Osterode am Harz, Germany) and an Optima MAX-XP ultracentrifuge (Beckman Coulter, Brea, CA, USA) for sample separation; a Nano-ZS laser particle size analyzer (Malvern Instruments Ltd., Malvern, UK) for particle size and ζ-potential measurements; a LUMisizer stability analyzer (LUM GmbH, Berlin, Germany) for stability analysis; a TSK-gel G-5000PWXL gel filtration column (Tosoh Corporation, Tokyo, Japan) and an Alliance 2695 HPLC system (Waters Corporation, Milford, CT, USA) for gel permeation chromatography (GPC) analysis; an HY300 constant-temperature oven (Shanghai Yiheng Scientific Instrument Co., Ltd., Shanghai, China) for sample drying; and an FA2004 electronic balance (accuracy 0.0001 g; Shanghai Precision Scientific Instrument Co., Ltd., Shanghai, China) for weighing. Ultrapure water was prepared using a system from Nanjing Eped Technology Co., Ltd. (Nanjing, China.)

### 2.3. Preparation of Acidified Dairy Beverages

A specific amount of skim milk powder (3 g) was accurately weighed and dispersed in 100 mL of deionized water, followed by stirring at room temperature for 1 h to ensure complete hydration. The required amount of SSPS stabilizer was dissolved in deionized water at 70 °C and stirred on a magnetic stirrer at 70 °C for 30 min to achieve full hydration. After cooling both solutions to room temperature, they were mixed and stirred for 10 min. The pH of the mixture was adjusted to the target value (pH 3–7) using a 30% citric acid solution or NaOH as needed, followed by an additional 10 min of stirring to ensure uniform pH throughout the beverage. The mixture was then heated to 60 °C and homogenized at a pressure of 200 bar. After homogenization, the solution was pasteurized in a 65 °C water bath for 30 min to obtain the final product. The resulting acidified dairy beverage contained 1.0% protein [11].

### 2.4. Measurement of Centrifugal Sedimentation Rate

A clean 10 mL centrifuge tube was weighed (*m*_0_), after which 8 mL of acidified dairy beverage was added and the tube was weighed again (*m*_1_). T The sample was centrifuged at 4 °C for 10 min at relative centrifugal forces (RCF) of approximately 2870× *g*, 8790× *g*, and 18,000× *g*. After centrifugation, the supernatant was carefully discarded, and the tube was inverted for 5 min before being weighed (*m*_2_). The sedimentation rate was then calculated as follows [12]:

$$Precipitation\;rate\left(\%\right)=\frac{m_{2}-m_{0}}{m_{1}-m_{0}}\times 100$$

### 2.5. Measurement of Particle Size and ζ-Potential

The particle size distribution and ζ-potential of proteins in acidified dairy beverages were determined using a Malvern laser particle size analyzer. The measurement parameters were set as follows: sample refractive index, 1.590; dispersant (water) refractive index, 1.330; and test temperature, 25 °C [13]. To minimize the influence of multiple scattering at high sample concentrations, the dairy beverage was diluted 1:100 with 0.02 mol/L citrate buffer adjusted to the same pH as the sample prior to measurement.

### 2.6. Measurement of Viscosity

The viscosity of the acidified dairy beverages was measured using a HAAKE MARS III rheometer equipped with a cone–plate geometry (60 mm diameter, 1° cone angle). The shear rate was set in the range of 0.1–100 s^−1^, and the measurement temperature was maintained at 25 °C. All samples were analyzed within 24 h after preparation of the acidified dairy beverages [13].

### 2.7. LUMisizer Stability Analysis and Determination Method

The stability of dairy beverages was evaluated using a LUMisizer stability analyzer. The measurement parameters were set as follows: centrifugation speed, 4000 r/min; number of profiles, 560; time interval between profiles, 15 s; optical factor, 1.0; measurement temperature, 25 °C; light source, 470 nm; and total measurement time, 2 h 20 min [14]. Gel permeation chromatography (GPC) was further applied to analyze the supernatant components. Separation was performed on a TSK-GEL 5000PWXL column (7.8 mm × 300 mm, Tosoh Corporation, Japan) equipped with both a refractive index (RI) detector and a UV detector, using a Waters HPLC system. The column temperature was maintained at 40 °C, and the injection volume was 30 μL. The mobile phase was 0.1 mol/L phosphate buffer (pH 6.8) at a flow rate of 0.6 mL/min. The polysaccharide and protein contents in the supernatant were determined based on the RI and UV signals.

For quantitative analysis, standard calibration curves were constructed with the concentrations of SSPS and pectin as the *x*-axis and the corresponding peak areas as the *y*-axis. The standard curve for SSPS was y = 326,886x (R^2^ = 0.9990), and that for pectin was y = 307,373x (R^2^ = 0.9995).

### 2.8. Data Analysis

All experiments were performed in triplicate or more, and the results are expressed as mean ± standard deviation. Statistical analysis was performed using one-way ANOVA with Tukey’s test in SPSS 2.0, with *p* < 0.05 considered significant.

## 3. Results and Discussion

### 3.1. Effect of pH on the Centrifugal Sedimentation Rate of Formulated Dairy Beverages

**Figure 1:**
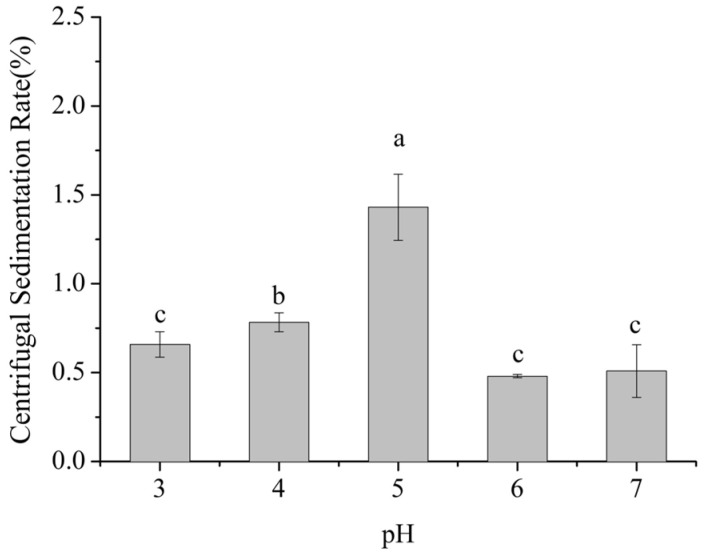
Effect of pH on the centrifugal sedimentation rate of formulated dairy beverages. Different letters indicate significant differences among samples (*p* < 0.05).

Centrifugal sedimentation rate is a key indicator of the propensity of particles to settle in dairy beverage systems. A higher sedimentation rate indicates stronger aggregation and sedimentation of milk proteins and other particles, indicating lower system stability. As shown in Figure 1, within the pH range of 3–7, the centrifugal sedimentation rate of the dairy beverages exhibited a significant trend of first increasing and then decreasing with pH (*p* < 0.05).

Within the pH range of 3–5, the centrifugal sedimentation rate gradually increased from 0.7% to 1.5%, reaching a maximum at pH 5, which was 114% higher than at pH 3. From pH 5 to 7, the sedimentation rate decreased from 1.5% to 0.5%, with the value at pH 7 being 67% lower than that at pH 5. No significant difference in sedimentation rate was observed between pH 6 and pH 7 (*p* > 0.05).

This phenomenon is closely related to the pIs of milk proteins, including caseins (pI ≈ 4.6) and whey proteins (pI ≈ 5.1–5.3). Caseins, which account for approximately 80–82% of skim milk proteins, have a pI near pH 4.6, whereas whey proteins, mainly α-lactalbumin and β-lactoglobulin and accounting for about 20% of the total, exhibit pI values in the range of pH 5.1 to 5.3. When the system pH is around 5.0, it nears the isoelectric regions of both caseins and whey proteins. At this point, the net surface charge of protein molecules is minimized, substantially reducing electrostatic repulsion and allowing intermolecular van der Waals interactions to predominate. Consequently, protein molecules readily aggregate into larger particles, which sediment more easily under centrifugation, resulting in a pronounced increase in the sedimentation rate. [15,16].

When the pH increased to 6–7, approaching the near-neutral range, the system moved away from the pIs of milk proteins. Casein molecules regained negative charges, which enhanced electrostatic repulsion between them. Simultaneously, SSPS molecules, rich in carboxyl groups (-COOH), were fully dissociated into -COO^−^ under neutral conditions. The negatively charged SSPS molecules adsorbed onto the surface of milk protein particles through electrostatic interactions, forming a polysaccharide adsorption layer approximately 10–15 nm in thickness. This adsorption layer not only strengthened the electrostatic repulsion between particles but also hindered collisions and aggregation of milk protein particles through steric effects, thereby significantly reducing the centrifugal sedimentation rate and maintaining system stability [17,18].

When the pH decreased to 3–4, although the system pH was below the pI of caseins (pH 4.6) and milk protein molecules carried positive charges, the dissociation state of SSPS was altered. The pIs of SSPS is approximately pH 3.2, and at pH 3–4, SSPS molecules retained partial negative charges, with ζ-potential values ranging from −5 to −15 mV. These negatively charged SSPS molecules interacted electrostatically with positively charged milk proteins to form stable SSPS–milk protein complexes. Moreover, under low pH conditions, casein micelles gradually dissolved, and the hydrophilic segments of κ-casein, such as the glycan chains, collapsed [19]. This structural change facilitated stronger interactions between SSPS molecules and the hydrophobic domains of caseins, further enhancing the stability of the complexes. Consequently, the centrifugal sedimentation rate was significantly lower than that observed at pH 5 [17].

### 3.2. Effect of pH on Particle Size and ζ-Potential of Formulated Dairy Beverages

#### 3.2.1. Particle Size Distribution

**Figure 2:**
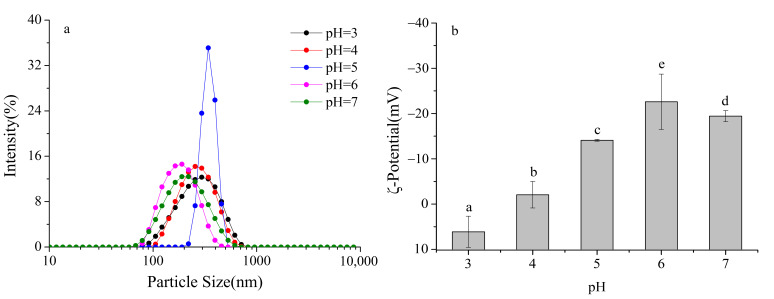
Effect of pH on the particle size distribution and ζ- potential of formulated dairy beverages. (**a**) particle size distribution, (**b**) ζ- potential. Different letters indicate significant differences among samples (*p* < 0.05).

Particle size distribution provides a direct measure of the dispersion state of particles in dairy beverage systems. Smaller and more uniform particle sizes are indicative of better dispersibility and greater system stability. As shown in Figure 2a, the dairy beverage systems containing 0.4% SSPS exhibited a monomodal particle size distribution across pH 3–7, with no obvious secondary peaks. This indicates that the systems maintained good dispersion stability under all tested pH conditions, demonstrating the stabilizing effect of SSPS in preventing excessive aggregation within the formulations.

Further analysis of the particle size distribution curves and peak positions revealed that at pH 6–7, the peaks shifted farthest to the left, with volume-weighted mean diameters (D[4,3]) of 182.3 nm at pH 6) and 195.6 nm at pH 7. The distribution widths (Span values) were also the smallest, at 0.32 and 0.35, respectively. These results demonstrate that in this pH range, milk protein particles were smaller, more uniformly distributed, and exhibited optimal dispersibility.

At pH 5, the particle size distribution curve shifted markedly to the right, with D[4,3] increasing to 356.7 nm and the Span value rising to 0.68, indicating a significantly broader distribution and the presence of numerous large aggregates in the system. Notably, no visible sedimentation was observed under static conditions, and significant sedimentation occurred only after centrifugation. This suggests that SSPS molecules adsorbed onto the surfaces of protein aggregates, providing steric hindrance that temporarily delayed sedimentation, although the formation of aggregates still led to a substantial increase in particle size.

At pH 3–4, the particle size distribution curves gradually shifted to the left, with D[4,3] decreasing from 289.5 nm at pH 4 to 210.8 nm at pH 3, and the Span value decreasing from 0.56 to 0.41, indicating progressively smaller and more uniform particles. This trend can be attributed to the dissolution of casein micelles under acidic conditions: phosphate groups on casein molecules bind protons, leading to micelle disintegration and exposure of the hydrophobic C-terminal segments of κ-casein. The hydrophobic groups of SSPS molecules, such as methyl groups and aromatic rings, interact with these exposed hydrophobic segments through hydrophobic interactions, while the hydrophilic groups of SSPS, such as hydroxyl and carboxyl groups, orient toward the aqueous phase, forming stable core–shell complexes. This interaction effectively reduced particle size and enhanced system stability [20,21].

#### 3.2.2. ζ-Potential

ζ-Potential reflects the surface charge of particles, with larger absolute values indicating stronger electrostatic repulsion and higher system stability. When the absolute value of ζ-potential is less than 20 mV, electrostatic repulsion becomes insufficient and particles tend to aggregate [22]. As shown in Figure 2b, the ζ-potential of SSPS–milk protein complexes exhibited a significant and systematic variation under different pH conditions.

At pH 7, the ζ-potential of the complexes was −19.5 mV, indicating a relatively high absolute value, strong electrostatic repulsion between particles, and high system stability. At this pH, the carboxyl and phosphate groups on the surface of milk protein particles were fully dissociated, imparting negative charges. The carboxyl groups of SSPS molecules were also fully dissociated into -COO^−^, contributing additional negative charges. Similarly, at pH 6, the ζ-potential remained high, suggesting that both SSPS and milk proteins carried strong negative charges, which maintained strong electrostatic repulsion and good dispersion stability. Electrostatic repulsion between the negatively charged SSPS and protein particles inhibited aggregation, while SSPS adsorption further increased the surface charge density, resulting in a higher absolute ζ-potential. Within the pH range of 4–7, as pH decreased, the ζ-potential of the complexes gradually increased from −19.5 mV at pH 7 to −2.4 mV at pH 4, reflecting a continuous reduction in negative charges. This is because the increased proton concentration neutralized the negative groups on the milk protein surface (e.g., -COO^−^, -PO_4_^2−^), reducing the surface charge density. Simultaneously, the dissociation of carboxyl groups in SSPS decreased, leading to fewer negative charges in the complexes. At pH 4.6, corresponding to the pI of caseins, the ζ-potential dropped to −5.8 mV, with an absolute value below 10 mV, indicating very weak electrostatic repulsion. This accounts for the formation of large aggregates observed around pH 5.

When the pH fell below 4.6, the negative groups on the surface of milk proteins were fully protonated, and the protein molecules began to carry positive charges. At this stage, SSPS molecules still carried partial negative charges (ζ-potential of −2.4 mV at pH 4), allowing the positively charged milk proteins to interact with negatively charged SSPS through electrostatic attraction. This interaction partially neutralized the protein surface charges, further reducing the overall negative charge of the complexes. At pH below 3.2, corresponding to the isoelectric point of SSPS, the carboxyl groups of SSPS were fully protonated and no longer carried negative charges. Instead, the molecules became positively charged due to protonation of the small amount of amino groups. Under these conditions, both milk proteins and SSPS carried positive charges, and the ζ-potential of the complexes became positive (+5.2 mV at pH 3), indicating limited electrostatic repulsion between particles. Moreover, SSPS and milk proteins formed stable complexes through hydrophobic interactions, which helped maintain a certain degree of stability, as reflected by the relatively low centrifugation sedimentation rate.

### 3.3. Effect of pH on the Viscosity of Formulated Dairy Beverages

Viscosity is a key indicator reflecting the flow behavior and internal structure of dairy beverage systems. Excessively high viscosity can result in a thick and unpalatable texture, negatively affecting the drinking experience. Abnormal changes in viscosity are often associated with particle aggregation or the formation of network structures within the system. As shown in Table 1, at a shear rate of 10 s^−1^, the viscosities of dairy beverages at different pH values differed significantly (*p* < 0.05).

At pH 6–7, the viscosities of the dairy beverages were the lowest, measuring 4.03 mPa·s at pH 6 and 4.56 mPa·s at pH 7, with no significant difference between them (*p* > 0.05). This trend was consistent with the particle size results: in this pH range, milk protein particles were small and uniformly dispersed, and intermolecular interactions such as van der Waals forces and hydrogen bonding were relatively weak. Consequently, the system exhibited a loose internal structure and low viscosity, which corresponds to the “refreshing and easy-to-drink” texture desired in formulated dairy beverages [23,24]. At pH 5, viscosity reached a maximum of 6.83 mPa·s, representing a 69.5% increase compared with pH 6. This is attributed to the presence of a large number of sizeable SSPS–milk protein aggregates (mean particle size 356.7 nm) in the system. These aggregates were interconnected through hydrogen bonding and hydrophobic interactions, forming a loose network structure that hindered fluid flow and markedly increased viscosity. In addition, the presence of aggregates enhanced internal friction, further contributing to the viscosity rise. At pH 3–4, viscosity decreased gradually from 5.00 mPa·s at pH 4 to 4.78 mPa·s at pH 3. This decline resulted from the progressive dissolution of casein micelles under acidic conditions, which caused large aggregates to dissociate into smaller SSPS–milk protein complexes (particle size decreasing from 289.5 nm to 210.8 nm). The disruption of the internal network structure and the weakening of intermolecular interactions reduced flow resistance, leading to a gradual decrease in viscosity.

Correlation analysis revealed a significant positive relationship between the viscosity of dairy beverages and the volume-weighted mean diameter of SSPS–milk protein complexes (R^2^ = 0.92, *p* < 0.01), indicating that larger particle sizes were associated with higher viscosities. These results suggest that pH regulates system viscosity primarily by influencing the particle size of the complexes. Moreover, viscosity changes indirectly reflect system stability: excessively high viscosity, as observed at pH 5, is typically associated with the presence of numerous aggregates and poor stability, whereas moderate and stable viscosity, as observed at pH 6–7, corresponds to uniform particle dispersion and favorable system stability.

### 3.4. Effect of pH on the LUMisizer Stability of Formulated Dairy Beverages

**Figure 3:**
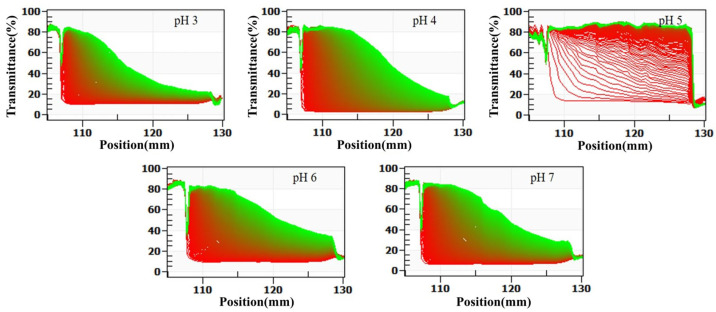
Effect of pH on the stabilization analysis of formulated dairy beverages by LUMisizer. (Green regions indicate stable dispersions with low sedimentation, whereas red regions correspond to faster sedimentation or less stable areas).

The LUMisizer stability analysis evaluates system stability by continuously monitoring changes in sample transmittance under centrifugation in real time. Compared with conventional centrifugation-based sedimentation measurements, this method offers faster and more accurate evaluation, with enhanced sensitivity and dynamic resolution. As illustrated in Figure 3, dairy beverages prepared at different pH values displayed distinct patterns of transmittance variation and final stability throughout the centrifugation process.

At pH 5, the system exhibited the most pronounced changes in transmittance. Within the first 5 min of centrifugation, the transmittance in the central region of the sample cell increased sharply from 15% to 50%. After 30 min, the transmittance in all regions except the bottom sediment layer exceeded 80%, and the transmittance profile displayed a clear stepwise increase. This pattern indicates the rapid sedimentation of a large number of particles and the formation of a clear boundary between the supernatant and sediment, reflecting the lowest stability of the system. Additionally, the stability index (TSI) calculated using SEPS software was 8.6 at pH 5, which was significantly higher than that of other pH groups (*p* < 0.05), further confirming the pronounced instability of the system under this condition.

At pH 6 and 7, the changes in transmittance during centrifugation were minimal. After 30 min, the transmittance increased only slightly, from 12% to 18% at pH 6 and from 14% to 22% at pH 7. The transmittance curves remained nearly identical to the initial curves, and no distinct phase separation was observed. The corresponding TSI values were 1.2 at pH 6 and 1.5 at pH 7, the lowest among all pH groups, indicating extremely slow particle sedimentation, high dispersibility, and optimal system stability.

At pH 3 and 4, the transmittance changes were intermediate between those observed at pH 5 and at pH 6 and 7. After 30 min of centrifugation, transmittance increased from 18% to 28% at pH 3 and from 16% to 35% at pH 4. The corresponding TSI values were 3.5 at pH 3 and 4.8 at pH 4. Although transmittance increased to some extent, the magnitude of change was much smaller than that observed at pH 5, and no distinct sedimentation layers were detected. These findings suggest a relatively slow particle sedimentation rate, with system stability higher than at pH 5 but lower than at pH 6 and 7.

The LUMisizer analysis results were in strong agreement with measurements of centrifugal sedimentation rate, particle size, ζ-potential, and viscosity. From the perspective of dynamic monitoring, these findings further confirmed that SSPS conferred the greatest stabilization of dairy beverages at pH 6 and 7. At pH 5, close to the isoelectric point of milk proteins, system stability was at its lowest. As the pH decreased to 3 and 4, stability gradually improved. This integrated validation provides a more comprehensive and reliable experimental basis for elucidating the role of pH in the stabilization of dairy beverage systems by SSPS.

## 4. Conclusions

In this study, a formulated dairy beverage containing 1.0% protein was used as a model system, with 0.4% SSPS as a stabilizer. The effects of pH (3–7) on system stability and the underlying mechanisms were systematically investigated through multiple indicators, including particle size, ζ-potential, viscosity, sedimentation rate, and LU-Misizer stability.

The results revealed a clear pH-dependent stability pattern: the lowest stability occurred at pH 5, due to proximity to the pIs of milk proteins, which reduced net charge, weakened electrostatic repulsion, and promoted the formation of large aggregates. At pH 6–7, both milk proteins and SSPS carried negative charges, and the combined effects of electrostatic repulsion and steric hindrance effectively prevented aggregation, resulting in the highest stability. At pH 3–4, partially positive proteins and negatively charged SSPS formed stable complexes, and the partial dissociation of casein micelles limited aggregate formation, contributing to moderate stability.

This study provides novel insights into the interaction mechanisms between SSPS and milk proteins across a broad pH range, highlighting the synergistic roles of electrostatic interactions, steric hindrance, and hydrophobic interactions in regulating system stability. From a practical perspective, the findings offer precise pH control parameters for industrial production, with pH 6–7 recommended as optimal to prevent sedimentation and phase separation. Moreover, the approach provides a reference for the application of other plant-derived polysaccharide stabilizers in formulated dairy beverages.

## Figures and Tables

**Table 1:** Effect of pH on the viscosity of formulated dairy beverages (shear rate = 10 s^−1^).

pH	3	4	5	6	7
Viscosity (mPa·s)	4.78 ± 0.12 b	5.00 ± 0.15 b	6.83 ± 0.20 a	4.03 ± 0.30 b	4.56 ± 0.13 b

Different letters within the same column indicate significant differences between the data (*p* < 0.05).

## Data Availability

The original contributions presented in this study are included in the article. Further inquiries can be directed to the corresponding author.
